# Xenograft assessment of predictive biomarkers for standard head and neck cancer therapies

**DOI:** 10.1002/cam4.387

**Published:** 2015-01-26

**Authors:** Andrew P Stein, Adam D Swick, Molly A Smith, Grace C Blitzer, Robert Z Yang, Sandeep Saha, Paul M Harari, Paul F Lambert, Cheng Z Liu, Randall J Kimple

**Affiliations:** 1 Department of Human Oncology, University of Wisconsin School of Medicine and Public HealthMadison, Wisconsin, 53706; 2 Department of Biostatistics, University of Wisconsin School of Medicine and Public HealthMadison, Wisconsin, 53706; 3 Department of Oncology, University of Wisconsin School of Medicine and Public HealthMadison, Wisconsin, 53706; 4 Department of Pathology, University of Wisconsin School of Medicine and Public HealthMadison, Wisconsin, 53706

**Keywords:** Head and neck cancer, HNSCC, HPV, predictive biomarkers, xenografts

## Abstract

Head and neck squamous cell carcinoma (HNSCC) remains a challenging cancer to treat with overall 5-year survival on the order of 50–60%. Therefore, predictive biomarkers for this disease would be valuable to provide more effective and individualized therapeutic approaches for these patients. While prognostic biomarkers such as p16 expression correlate with outcome; to date, no predictive biomarkers have been clinically validated for HNSCC. We generated xenografts in immunocompromised mice from six established HNSCC cell lines and evaluated response to cisplatin, cetuximab, and radiation. Tissue microarrays were constructed from pre- and posttreatment tumor samples derived from each xenograft experiment. Quantitative immunohistochemistry was performed using a semiautomated imaging and analysis platform to determine the relative expression of five potential predictive biomarkers: epidermal growth factor receptor (EGFR), phospho-EGFR, phospho-Akt, phospho-ERK, and excision repair cross-complementation group 1 (ERCC1). Biomarker levels were compared between xenografts that were sensitive versus resistant to a specific therapy utilizing a two-sample t-test with equal standard deviations. Indeed the xenografts displayed heterogeneous responses to each treatment, and we linked a number of baseline biomarker levels to response. This included low ERCC1 being associated with cisplatin sensitivity, low phospho-Akt correlated with cetuximab sensitivity, and high total EGFR was related to radiation resistance. Overall, we developed a systematic approach to identifying predictive biomarkers and demonstrated several connections between biomarker levels and treatment response. Despite these promising initial results, this work requires additional preclinical validation, likely involving the use of patient-derived xenografts, prior to moving into the clinical realm for confirmation among patients with HNSCC.

## Introduction

Head and neck squamous cell carcinoma (HNSCC) represents the eighth most common malignancy worldwide 1, and the overall 5-year survival rate is on the order of 50–60% 2. These patients often present with locoregionally advanced disease 3 and are treated with a combination of surgery, radiation, and chemotherapy. Notably, these therapies can induce serious acute and long-term consequences including mucositis, xerostomia, dysphagia, and others 4–6.

Over the past decade, human papillomavirus (HPV) has emerged as an important causative agent in a subset of HNSCCs 7,8, and patients with HPV-positive cancers demonstrate improved response to standard therapies 9–11. p16 immunohistochemistry (IHC) is used clinically as a surrogate for HPV positivity as p16 overexpression is mechanistically linked to HPV oncogene expression. In this manner, p16 represents a prognostic biomarker for HNSCC 12,13. Although prognostic biomarkers identify patients that are likely to see improved overall survival, these markers do not dictate treatment. On the other hand, predictive biomarkers are ones that could potentially guide therapy.

Despite research dedicated to identifying predictive biomarkers for HNSCC 14–16, none have been clinically validated. Current investigations include the relationship between excision repair cross-complementation group 1 (ERCC1) expression and cisplatin response 17–19 as well as levels of the epidermal growth factor receptor (EGFR) in relation to cetuximab sensitivity 4,20,21. Other groups have evaluated both EGFR and ERCC1 as they relate to radiation response 18,22,23. Few studies have examined the predictive potential of proteins downstream of EGFR (e.g., phospho-EGFR, pEGFR; phospho-Akt, pAkt; or phospho-ERK, pERK) in terms of cetuximab or radiation response. Additionally, as most studies attempt to identify biomarkers obtained from pretreatment biopsies, the utility of biomarkers obtained from posttreatment tumor samples, including circulating tumor cells, remains unclear.

Here, we report the investigation of specific biomarkers in relation to cisplatin (ERCC1), cetuximab (EGFR, pEGFR, pAkt, and pERK) and radiation (EGFR, pEGFR, pAkt, pERK, and ERCC1) treatments for six different HNSCC cell line xenografts. We performed quantitative IHC on tissue microarrays (TMAs) constructed from untreated (control) and posttreatment samples (collected at two early time points) to identify potential relationships between biomarker expression and therapeutic response. Overall, we sought to establish a robust system for identifying both pre- and posttreatment predictive biomarkers with the ultimate goal of guiding further preclinical validation studies and eventually improving patient care.

## Material and Methods

This study was carried out in accordance with the REMARK guidelines as applicable 24.

### Cell line xenografts

Established cell lines derived from patients with HPV-negative (UM-SCC1 and UM-SCC22B) or HPV-positive (UD-SCC2, UM-SCC47, UPCI-SCC90, and 93-VU-147T) HNSCC were used to generate xenografts as previously described 25. The source of each line and culture conditions are detailed in Table S1. All cell line identities were confirmed by short tandem repeat testing within 6 months of use. Mice were kept in the Association for Assessment and Accreditation of Laboratory Animal Care approved Animal Care Facility. Experiments were carried out in accordance with an animal protocol approved by our university.

### Therapeutic response

Tumor growth and response to cisplatin, cetuximab, and radiation were evaluated. Cells were amplified by in vitro culture 25, and xenografts were generated by injecting 1.5 million cells (in a 1:1 mixture of media [Dulbecco's Modified Eagle Medium with 10% fetal bovine serum and 1% penicillin/streptomycin] and matrigel [catalog #354230; BD Biosciences, Inc. San Jose, CA USA]) into the bilateral posterior flanks of 48 female Hsd:athymic Nude-*Foxn1*^*nu*^ mice (Harlan Laboratories, Madison, WI). Tumor volume was assessed twice weekly with Vernier calipers and calculated according to the equation *V* = (*π*/6) × (large diameter) × (small diameter)^2^. When tumor volumes reached an average of 200 mm^3^, mice were stratified into control (vehicle/mock radiation), cisplatin, cetuximab, or radiation-treated groups (*n* = 12 mice/24 tumors per group) such that all groups contained a range of similarly sized tumors. Treatment was initiated with vehicle control (0.95 normal saline), cisplatin (2 mg/kg), or cetuximab (0.2 mg/mouse) delivered by intraperitoneal (IP) injection twice weekly for 2 weeks. Radiation therapy (or mock treatment) was administered using an X-rad 320 biological irradiator (Precision X-ray, Inc. North Branford, CT USA) as four 2 Gy fractions over two consecutive weeks. After completing the treatment regimen, tumors were measured one to two times weekly until the majority of control tumors quadrupled in size. Tumor growth curves were generated using Graphpad Prism v6.0d. Growth curves for the HPV-positive xenografts have been previously reported 25. Importantly, the analysis presented here includes an additional experiment with UM-SCC47 xenografts with aggregate data presented.

### Tumor harvesting and TMAs construction

Tumors were harvested for formalin fixation and paraffin embedding (FFPE) at two early time points post treatment from mice in each group. These time points were defined with respect to the first radiation treatment (4 and 24 h after the initial radiation therapy or mock treatment). Two TMAs were constructed from the FFPE tumors. Hematoxylin and eosin (H&E)-stained slides from each tumor were reviewed and areas of SCC were marked for 1.0-mm core extraction. Cores were placed on the recipient microarray block using a Tissue Microarrayer (Estigen OU, Tartu, Estonia). All tumors were represented on the microarray by duplicate cores. The TMAs were sectioned (5 *μ*m) and H&E stains were carried out on the 30th section. Additional sections were used for IHC.

### Immunohistochemistry

Briefly, TMA slides were deparaffinized, rehydrated, and underwent heat-induced epitope retrieval in Tris-EDTA buffer (pH = 9.0). Next, endogenous peroxidase activity was blocked with a 0.3% hydrogen peroxide solution for 20 min, and nonspecific antibody interactions were blocked with 10% goat serum for 1 h. Slides were then incubated overnight at 4°C with a primary antibody dilution prepared in 1% goat serum. Primary antibodies utilized included EGFR, pEGFR, pAkt, pERK, and ERCC1 (Table S2). The following day anti-rabbit or anti-mouse horseradish peroxidase-conjugated secondary antibodies (Catalog #8114 and #8125; Cell Signaling Technology, Danvers, MA) were applied to the TMAs for 30 min at room temperature. This was followed by a 2-min development with 3,3′-diaminobenzidine (DAB) (#SK-4100; Vector Laboratories, Burlingame, CA) and 1-min counterstain with hematoxylin. Finally, slides were dried in a 60°C oven, dipped in fresh xylenes, and coverslipped with Cytoseal XYL (Thermo Fisher Scientific, Waltham, MA) and a number 1.5 coverslip. A no primary antibody control slide was stained with each set of TMAs to ensure the specificity of the staining (Fig. S1A).

The pEGFR and pAkt primary antibodies were also mixed with a pan-cytokeratin primary antibody (Table S2). TMAs stained with these primary antibody mixtures were first developed with DAB as described above (for pEGFR or pAkt). This was followed by a 30-min incubation with an anti-mouse alkaline phosphatase-linked secondary antibody (#MALP521; Biocare Medical, Concord, CA) and 2-min development with the Warp Red Chromogen Kit (#WR806; Biocare Medical) to detect pan-cytokeratin. After both primary antibodies were developed, the slides were counterstained, dried, and coverslipped.

### TMAs analysis

TMA slides were scanned using the Vectra System and analyzed by the inForm Software v1.4.0 (PerkinElmer, Waltham, MA). Single color control slides (UM-SCC47 control tumors stained only for hematoxylin, DAB, or Warp Red) were also scanned by Vectra and used to generate a spectral library using Nuance v3.0.0 software. As described previously, this library defines the spectral characteristics of each chromogen and allows for the unmixing and quantitation of each chromogen from the multicolored TMAs 26.

Automated imaging was performed on all cores in the TMA, with two 20x images obtained per core. An image analysis algorithm was then developed independently for each biomarker (EGFR, pEGFR, pAkt, pERK, and ERCC1) within one xenograft experiment. Each algorithm was generated in a few key steps by two of the authors (A. P. S. and C. Z. L.): image selection, tissue segmentation, and cell segmentation. First, one-third to one-half of the images from a specific experiment (depending on the heterogeneity of the tissue) were loaded into the inForm Software as the training images. Then, the system was taught to recognize two discrete tissue categories: SCC and other (necrosis, cystic change, etc.). Finally, cell segmentation was performed on the training images by identifying nuclei based on the hematoxylin stain. The algorithm was then applied to all images within that specific experiment (batch analysis). This process was repeated to generate data for each biomarker within every experiment.

As a quality control measure, all images were reviewed after batch analysis and cores with poor tissue segmentation were excluded. The final output from the inForm Software was the DAB mean optical density (MOD) (continuous value from 0 to 1) within the SCC tissue category of each image as well as the nuclear fraction of the SCC. In this analysis, DAB MOD represents protein (biomarker) expression and can thereby be utilized to compare biomarker levels between different experiments. SCC tissue data were utilized for EGFR, pEGFR, pAkt, and pERK as these proteins are found in both the membrane/cytoplasm and nucleus, while nuclear data were used for ERCC1 as it is solely expressed in this compartment. Since each tumor was represented on the TMA in duplicate and two images were obtained for each core, four DAB MOD values were obtained per tumor. A weighted average from the four images (based on the number of SCC pixels identified in each image) was calculated so each tumor ended up with a single DAB MOD value per biomarker.

We wanted to determine if pan-cytokeratin would improve the segmentation accuracy of the TMAs, so we carried out dual staining with pan-cytokeratin and pEGFR or pAkt. Based on our review of the tissue segmentation performance after batch analysis, we determined that there was no difference in the overall segmentation accuracy with or without pan-cytokeratin. For this reason, only two of the five biomarkers analyzed in this work utilized pan-cytokeratin as an additional tumor marker.

### Statistical analysis

Statistical analyses were carried out with the goal of identifying which xenografts were sensitive or resistant to specific treatments as well as to determine if any pre- (i.e., vehicle control or mock radiation) or posttreatment biomarkers were associated with therapeutic response. At the start of treatment, tumor volumes in each group were normalized to an average of 100%. Tumors were then measured until most control tumors quadrupled in size. To evaluate the sensitivity of each xenograft to cisplatin, cetuximab, and radiation, we compared the mean volumes between the control and treated tumors at the final three time points using two-sample t-tests with equal standard deviations. A *P*-value less than 0.05 was considered statistically significant. Xenografts with at least two of three significant *P*-values were deemed sensitive to the specific treatment, while those not meeting this criterion were considered resistant.

Specific biomarkers were examined in relation to each treatment regimen: cisplatin (ERCC1), cetuximab (EGFR, pEGFR, pAkt, and pERK), and radiation (EGFR, pEGFR, pAkt, pERK, and ERCC1). For each experiment, untreated control tumors harvested at the two early time points were utilized as our baseline or pretreatment equivalents (Fig. S1B). To evaluate the potential associations between untreated biomarker levels and therapeutic response, the DAB MOD values from control tumors were compared for xenografts that were sensitive versus resistant to a specific treatment. Next, we determined if there was any relationship between posttreatment biomarker expression and therapeutic response. For an individual experiment, DAB MOD values from the treated tumors were normalized to their respective untreated (control) tumors. In this manner, posttreatment biomarker expression was represented as a percentage of the untreated values. These normalized posttreatment values were pooled and compared for xenografts that were sensitive or resistant to a specific treatment. For all biomarker analyses, the sensitive and resistant groups were compared utilizing a two-sample t-test with equal standard deviations, and a *P*-value less than 0.05 was considered statistically significant. All statistical analyses in this manuscript were carried out using Graphpad Prism v6.0d.

## Results

### Cisplatin-sensitive xenografts demonstrate lower baseline ERCC1

**Figure 1:**
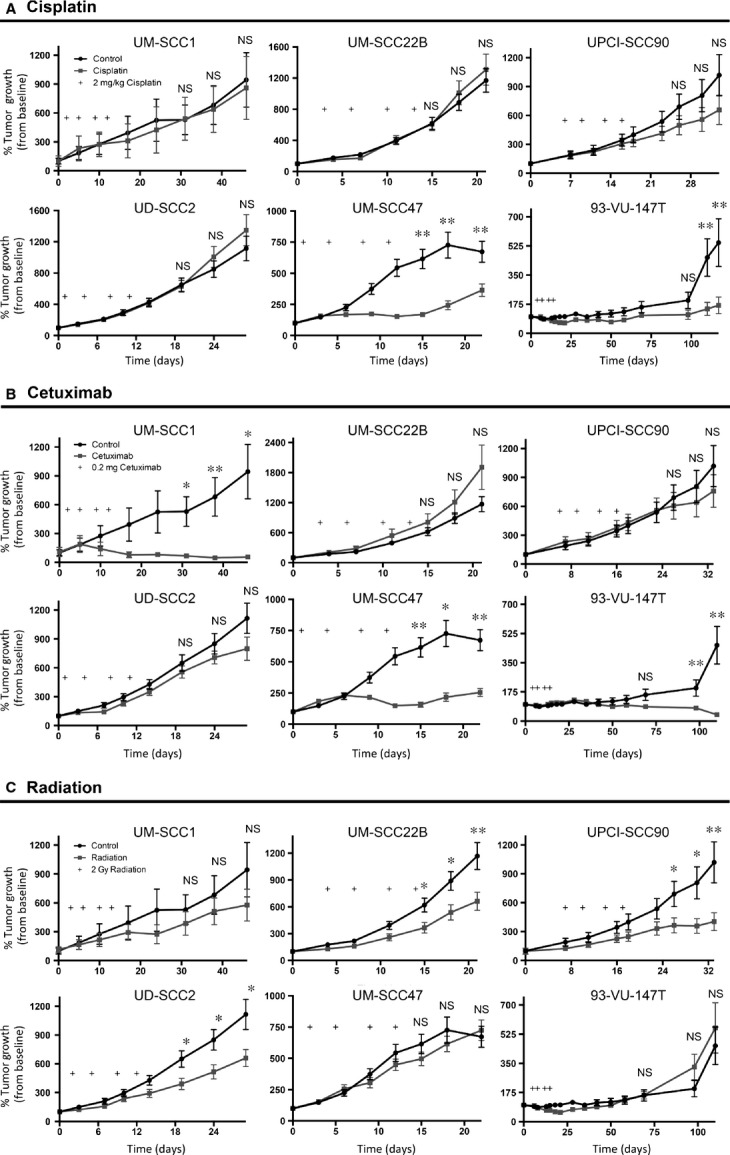
Tumor growth curves after cisplatin, cetuximab, and radiation treatments. (A–C) Tumor growth curves generated after treatment with cisplatin (A), cetuximab (B), and radiation (C) for each xenograft. Sensitivity was defined as a statistical difference in mean tumor volume between control and treated tumors for at least 2 of the final 3 time points (*P < 0.05, **P < 0.01, NS, not significant).

**Figure 2:**
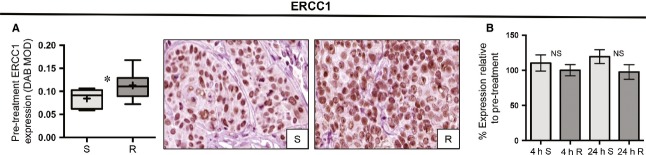
Relationship between baseline and posttreatment ERCC1 expression and cisplatin therapy. (A) Box plots and representative IHC images comparing baseline nuclear ERCC1 expression for the sensitive (S) versus resistant (R) xenografts (DAB MOD: DAB mean optical density; *P < 0.05). (B) Bar graphs with standard error of the mean depicting the percentage change of ERCC1 expression (relative to baseline, *n* = 24) in the sensitive versus resistant groups at 4 h (4 h, *n* = 12) and 24 h (24 h, *n* = 12) post treatment. NS, not significant; IHC, immunohistochemistry; ERCC1, excision repair cross-complementation group 1; DAB, 3,3′-diaminobenzidine; MOD, mean optical density.

As demonstrated in Figure1A, two xenografts were sensitive to cisplatin while four were resistant. Baseline ERCC1 expression (Table1) for the sensitive xenografts was significantly lower than the resistant cohort (*P* = 0.020, Fig.2A). At the posttreatment time points, no difference in the percentage change from baseline ERCC1 levels emerged for the sensitive compared to resistant xenografts (Figs.2B and S2).

**Table 1:** Pretreatment expression of specific biomarkers for xenografts that were sensitive versus resistant to cisplatin, cetuximab, and radiation treatments

Biomarker	Sensitive xenografts Average DAB MOD (95% CI)	Resistant xenografts Average DAB MOD (95% CI)	*P*-value
Cisplatin			
ERCC1	0.085 (0.068, 0.101)	0.113 (0.097, 0.129)	0.020*
Cetuximab			
EGFR	0.191 (0.164, 0.218)	0.114 (0.086, 0.141)	<0.001**
pEGFR	0.069 (0.052, 0.087)	0.071 (0.048, 0.094)	0.905
pAkt	0.010 (0.006, 0.013)	0.058 (0.037, 0.079)	<0.001**
pERK	0.023 (0.017, 0.029)	0.038 (0.020, 0.057)	0.093†
Radiation			
EGFR	0.114 (0.086, 0.141)	0.191 (0.164, 0.218)	<0.001**
pEGFR	0.071 (0.048, 0.094)	0.069 (0.052, 0.087)	0.905
pAkt	0.058 (0.037, 0.079)	0.010 (0.006, 0.013)	<0.001**
pERK	0.038 (0.020, 0.057)	0.023 (0.017, 0.029)	0.093†
ERCC1	0.118 (0.102, 0.135)	0.084 (0.071, 0.097)	0.002**

DAB, 3,3′-diaminobenzidine; MOD, mean optical density.

^†^*P* < 0.10, *P < 0.05, **P < 0.01.

### Intracellular signaling is associated with cetuximab response

**Figure 3:**
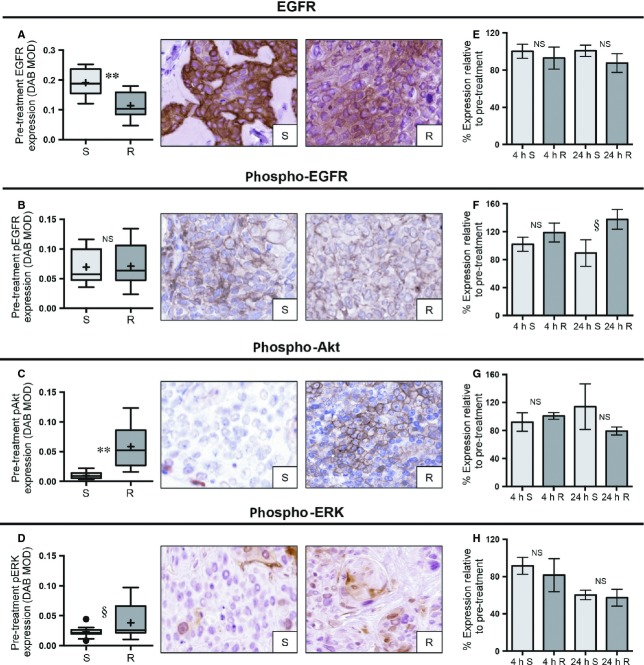
Relationship between baseline and posttreatment biomarker expression and cetuximab response. (A–D) Box plots and representative IHC images comparing the baseline expression of EGFR, pEGFR, pAkt, and pERK for sensitive (S) versus resistant (R) xenografts (DAB MOD: DAB mean optical density; ^§^*P* < 0.10, *P < 0.05, **P < 0.01). (E–H) Bar graphs with standard error of the mean depicting the percentage change for the expression of each indicated biomarker (relative to baseline, *n* = 24) in the sensitive versus resistant groups at 4 h (4 h, *n* = 12) and 24 h (24 h, *n* = 12) post treatment. NS, not significant; IHC, immunohistochemistry; EGFR, epidermal growth factor receptor; DAB, 3,3′-diaminobenzidine; MOD, mean optical density.

UM-SCC1, UM-SCC47, and 93-VU-147T were sensitive to cetuximab, while UD-SCC2, UM-SCC22B, and UPCI-SCC90 displayed resistance to this antibody (Fig.1B). Baseline expression of EGFR, pEGFR, pAkt, and pERK were compared between the sensitive and resistant xenografts (Table1, Fig.3A–D). The sensitive group had modestly higher expression of EGFR compared to the resistant xenografts (1.5-fold higher, *P* < 0.001), but there was no difference in pEGFR expression (*P* = 0.905). Sensitive xenografts had sixfold lower pAkt levels (*P* < 0.001), while pERK expression in the sensitive group trended lower, but did not reach significance (1.6-fold lower, *P* = 0.093).

Posttreatment biomarker expression was compared between the sensitive and resistant groups (Figs.3E–H and S3A–D). The only biomarker approaching significance was pEGFR at 24 h (*P* = 0.068), with the sensitive group demonstrating decreased pEGFR expression relative to control (89.3% (95% CI: 36.1%, 142.5%) while the resistant group had a higher level of pEGFR (137.7% (95% CI: 101.4%, 173.9%). Interestingly, at the 24-h time point, significant decreases in pERK expression relative to baseline were demonstrated in both the sensitive and resistant xenografts, but there was no difference in the percentage change between these groups (*P* = 0.650).

### Radiation response and predictive biomarkers

**Figure 4:**
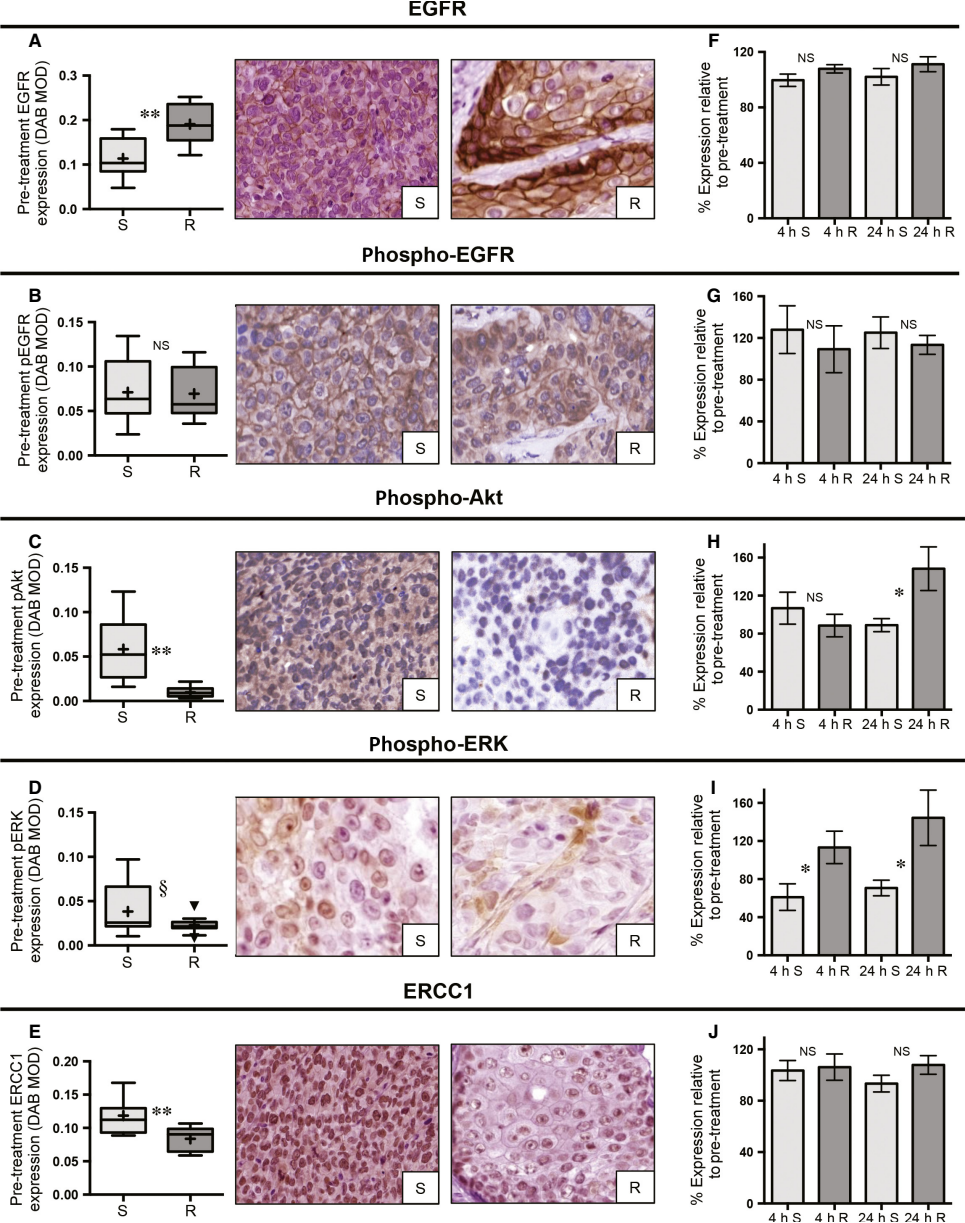
Relationship between baseline and posttreatment biomarker expression and radiation response. (A–E) Box plots and representative IHC images comparing the baseline expression values for the sensitive (S) versus resistant (R) groups with respect to EGFR, pEGFR, pAkt, pERK, and nuclear ERCC1 (DAB MOD: DAB mean optical density; ^§^*P* < 0.10, *P < 0.05, **P < 0.01). (F–J) Bar graphs with standard error of the mean depicting the percentage change for the expression of each indicated biomarker (relative to baseline, *n* = 24) in the sensitive versus resistant groups at 4 h (4 h, *n* = 12) and 24 h (24 h, *n* = 12) post treatment. NS, not significant; IHC, immunohistochemistry; EGFR, epidermal growth factor receptor; ERCC1, excision repair cross-complementation group 1; DAB, 3,3′-diaminobenzidine; MOD, mean optical density.

UD-SCC2, UM-SCC22B, and UPCI-SCC90 were classified as sensitive to radiation, while UM-SCC1, UM-SCC47, and 93-VU-147T were determined to be resistant (Fig.1C). Baseline levels of EGFR, pEGFR, pAkt, pERK, and ERCC1 were determined for xenografts that were sensitive or resistant to radiation (Table1, Fig.4A–E). EGFR was significantly higher in the resistant as compared to sensitive xenografts (*P* < 0.001), but there was no difference in pEGFR expression between these groups (*P* = 0.905). Although the sensitive group had lower total EGFR, they had almost sixfold greater expression of pAkt as compared to the resistant xenografts (*P *< 0.001). Moreover, although not statistically significant, there was a trend towards higher pERK expression in the sensitive versus resistant xenografts (*P* = 0.093). Finally, there was 1.4-fold higher ERCC1 expression in the sensitive as compared to resistant xenografts (*P* = 0.002).

We also determined whether there were any differences in posttreatment expression at 4 and 24 h between the sensitive and resistant groups (Figs.4F–J and S4A–E). No significant changes emerged with respect to posttreatment expression of EGFR or pEGFR. For pERK, there was a significant decrease (relative to baseline) at both 4 and 24 h for the sensitive versus resistant groups (*P* = 0.045 and 0.027, respectively). Although there was no difference in pAkt expression at 4 h, by 24 h the sensitive group had a significant decline in expression (*P* = 0.025). For ERCC1, there were no significant changes in posttreatment biomarker expression at 4 or 24 h (*P* = 0.839 and 0.169, respectively).

### HPV-positive xenografts

Since HPV-positive HNSCC represents a distinct clinical entity 7, we examined whether the baseline biomarker relationships exhibited above also related to HPV-positive xenografts alone (Table2). For cisplatin treatment, 93-VU-147T and UM-SCC47 were sensitive while UD-SCC2 and UPCI-SCC90 were resistant (Fig.1A). Consistent with the overall trend, ERCC1 expression was significantly lower in the sensitive compared to resistant group (*P* = 0.012). The HPV-positive xenografts had the same response to cetuximab as they did to cisplatin (Fig.1B). Baseline EGFR and pAkt expression upheld the same relationship described above: EGFR was significantly higher in the sensitive group (*P* = 0.029) while pAkt had roughly fivefold lower expression in the sensitive cohort (*P* = 0.006). There was no relationship between pERK (*P* = 0.642) or pEGFR (*P* = 0.927) levels and cetuximab response.

**Table 2:** Pretreatment expression of specific biomarkers for sensitive versus resistant HPV-positive xenografts with respect to cisplatin, cetuximab, and radiation treatments

Biomarker	Sensitive xenografts Average DAB MOD (95% CI)	Resistant xenografts Average DAB MOD (95% CI)	*P*-value
Cisplatin			
ERCC1	0.085 (0.068, 0.100)	0.122 (0.096, 0.148)	0.012*
Cetuximab			
EGFR	0.185 (0.146, 0.225)	0.133 (0.101, 0.165)	0.029*
pEGFR	0.082 (0.061, 0.104)	0.081 (0.046, 0.115)	0.927
pAkt	0.013 (0.008, 0.017)	0.060 (0.026, 0.094)	0.006**
pERK	0.023 (0.014, 0.032)	0.021 (0.015, 0.026)	0.642
Radiation			
EGFR	0.133 (0.101, 0.165)	0.185 (0.146, 0.225)	0.029*
pEGFR	0.081 (0.046, 0.115)	0.082 (0.061, 0.104)	0.927
pAkt	0.060 (0.026, 0.094)	0.013 (0.008, 0.017)	0.006**
pERK	0.021 (0.015, 0.026)	0.023 (0.014, 0.032)	0.642
ERCC1	0.122 (0.096, 0.148)	0.085 (0.068, 0.100)	0.012*

DAB, 3,3′-diaminobenzidine; MOD, mean optical density; HPV, human papillomavirus.

*P < 0.05, **P < 0.01.

The radiation response for the HPV-positive xenografts was opposite that of cisplatin and cetuximab: 93-VU-147T and UM-SCC47 were resistant, while UD-SCC2 and UPCI-SCC90 were sensitive (Fig.1C). EGFR was significantly higher in the resistant group (*P* = 0.029), while pAkt was lower in the resistant xenografts (*P* = 0.006). There was no difference in baseline expression for pERK (*P* = 0.642) or pEGFR (*P* = 0.927) between the sensitive and resistant groups. Finally, as was found for all six xenografts, ERCC1 was 1.4-fold higher in the sensitive versus resistant cohorts (*P* = 0.012).

## Discussion

Identifying reliable predictive biomarkers would be a valuable tool for adapting oncologic treatments to each individual patient, ideally resulting in fewer side effects, expedited patient recovery and improved cure rates. Despite efforts to identify such markers for HNSCC (reviewed in 15,16,27), none have been validated to date. While most biomarker studies rely on retrospective analysis of heterogeneously treated patient samples, we chose to pursue a different approach. By treating the same xenograft multiple times, these mouse models enabled the assessment of an average response. In a clinical study from which biomarker identification is often performed, the treatment outcome of each patient can only be investigated once. Using mouse models we were able, in essence, to “enroll” a subject on multiple arms of a given study. Furthermore, assessment of outcome in clinical studies introduces a number of potential biases that may cloud biomarker assessment including death from other causes, heterogeneous disease stages, inaccurate diagnosis, subsequent treatments received, and much more. Investigating the same six xenografts’ response to each of three therapies: cisplatin, cetuximab, and radiation, provided a controlled environment in which to relate tumor responses to the pre- and posttreatment expression of five potential predictive biomarkers: EGFR, pEGFR, pAkt, pERK, and ERCC1.

Cisplatin remains a commonly used chemotherapy agent for HNSCC, and although it provides therapeutic efficacy for a subset of patients 28, it can also induce major side effects in others without much clinical benefit. Therefore, it would be valuable to have a predictive biomarker to identify patients likely to respond to this agent. One potential marker is ERCC1, an essential protein in the nucleotide excision repair pathway, which removes DNA lesions caused by cisplatin. Low ERCC1 expression has been related to improved survival and better response to platinum therapy 19,29,30. We assayed the relative level of ERCC1 in our cisplatin-sensitive and -resistant xenografts using the highly specific monoclonal antibody 4F9, thereby avoiding potential nonspecificity that had been observed with the 8F1 clone 17,31. Our study confirmed that the cisplatin-responsive xenografts had significantly lower baseline ERCC1 expression than the resistant tumors. However, at the two early posttreatment time points, neither the sensitive nor resistant tumors had altered ERCC1 expression, suggesting that cisplatin did not affect the level of ERCC1 in these tumors during the time scale investigated.

Next, we analyzed the predictive potential of multiple proteins in the EGFR pathway (EGFR, pEGFR, pAkt, and pERK) in relation to cetuximab treatment, a commonly prescribed EGFR-targeting monoclonal antibody. Cetuximab and similar human epidermal growth factor receptor (HER) family targeting antibodies are thought to interact with tumor cells through two mechanisms: direct inhibition of ligand binding and thus downstream signaling, and induction of antibody-dependent cell-mediated cytotoxicity (ADCC), whereby natural killer cells and macrophages are recruited to the tumor cells and induce apoptosis 32,33. While previous studies have shown that total EGFR levels are not predictive of cetuximab sensitivity 20,34,35, we observed modestly higher total EGFR in the sensitive xenografts. However, we did not see a difference in pEGFR expression between sensitive and resistant xenografts, indicating that baseline EGFR activity did not differ between these groups. Interestingly, we observed that pAkt was significantly lower in sensitive tumors, with a similar trend for pERK. As these proteins are located downstream in multiple signaling pathways, they can be activated by HER family receptors aside from EGFR or by activating mutations in other upstream proteins 32,36,37. Considering pAkt and pERK serve as essential nodes of pro-proliferative activity, the potential utility of using these downstream molecules as predictive biomarkers is highlighted by our results.

Finally, we evaluated the relationship of all five biomarkers to radiation response. Consistent with previous reports 22,38, high total EGFR corresponded to radiation resistance, but no difference in the levels of the activated form, pEGFR, was seen between responders and nonresponders. Baseline expression of pAkt and pERK was both higher in the sensitive tumors prior to treatment. Additionally, we found that the expression of pAkt and pERK remained unchanged or increased in the resistant xenografts post treatment while we observed decreased expression for sensitive tumors. Consistent with other reports, this suggests sustained activation of these pro-growth signaling pathways is important in promoting radiation resistance 39,40. In this manner, targeting these downstream pathways may hold promise as an approach to improved radiosensitization. While a retrospective clinical study of patients with HPV-negative HNSCC-associated low ERCC1 with improved outcomes after surgery plus radiation 18, we found that high ERCC1 expression was related to increased radiation sensitivity. However, important differences in our study, including the use of a different primary antibody, potential adaptation of cell lines during in vitro culture and the presence of both HPV-positive and -negative tumor types, could help explain this apparent inconsistency.

Several groups have questioned the clinical relevance of cell lines and cell line xenografts since tumors generated from immortalized lines may not perfectly represent the primary tumor 41–44. We agree that this approach has limitations, but the methodology described in this study provided initial insight into the predictive potential of specific biomarkers and could be adapted to other model systems including patient-derived xenografts (PDXs) and patient samples. By forgoing any growth or adaptation to a tissue culture environment, PDXs appear to more closely replicate conditions in the original patient 41,45–47. Our group has already established HNSCC PDXs, are adding tumors to our repertoire, and are well positioned to move forward with this work 41,48. In any study of this type, investigators are also limited by the prespecified time points for posttreatment biomarker assessment. Our times were selected to identify immediate (4 h) and late effects (24 h). We also wanted to evaluate a time point that occurred close to when a typical fractionated treatment would be given in the clinical care of patients with HNSCC (i.e., 24 h). The resources necessary to investigate additional time points would have been considerable, so we only focused on the 4- and 24-h time points. It is possible that by focusing on these two times, we may have missed potentially important biomarker changes that occurred within minutes or days of therapy. We have previously described the UM-SCC47 cell line as sensitive to radiation, this difference can be explained by the assessment at an earlier time point in this study and the inclusion of additional control mice. Finally, while we only studied five potential predictive biomarkers, those chosen are mechanistically linked to the treatment regimens administered. In the future, our TMAs can be utilized to investigate additional potential biomarkers suggested by reports in the literature or our own in vitro screening. For example, disruptive mutations in p53 have been linked by other groups to radiation resistance 49,50.

Despite the above limitations, one advantage of the xenograft model we used was the ability to evaluate both pre- and posttreatment samples. Most retrospective studies assessing putative biomarkers only utilize pretreatment samples as, historically, tissue is not routinely obtained while patients are under treatment. Our analysis of posttreatment samples was significantly more successful than a prior clinical study in which we assessed posttreatment biopsies and demonstrated that multiple samples contained only necrotic tumor and were not analyzable 51. Due to the improved tissue quality, we obtained in this study, we were able to identify posttreatment changes in pAkt and pERK expression as potentially related to radiation response. These findings suggest that ongoing efforts to target these pathways may hold promise as potential radiation sensitizers in HNSCC. Importantly, transitioning posttreatment biomarker analyses to the clinical realm may not be as daunting as previously thought due to improvements in the ability to identify and isolate circulating tumor cells. In this manner, assessment of posttreatment tumor response may soon be as simple as drawing an extra vial of blood.

Considering HPV-positive HNSCCs comprise a growing proportion of all head and neck cancers 52, there is a strong interest in the oncology community to better understand and treat this subset of malignancies. While the four HPV-positive xenografts examined responded heterogeneously to treatment, the links between baseline biomarker levels and therapeutic response established in the broader study were largely maintained. For example, the cetuximab-sensitive xenografts displayed high total EGFR but low pAkt and pERK expression while the cisplatin-sensitive xenografts demonstrated low ERCC1 levels. Overall, these results highlight that while HPV-positivity has been clinically validated to be prognostic for improved overall survival, this subpopulation remains biologically heterogeneous and likely will require evaluation by an array of markers in order to be treated with optimal therapy. Relative changes in DAB-MOD values provided here would require standardization prior to being used in clinical applications, but are sufficient to support our intention of providing a proof-of-concept and provide a potential biological rationale for therapeutic responses. Future work should include assessment of biomarkers in tumors treated with combination therapy (cisplatin + radiation or cetuximab + radiation) and evaluation of biomarkers in additional preclinical systems.

Overall, we have attempted to take small steps forward in predictive biomarker identification for HNSCC. By analyzing the response of the same group of xenografts to different treatment modalities and linking those responses to a number of potential biomarkers, we have both confirmed previously described relationships (low ERCC1 associated with cisplatin sensitivity) as well as uncovered potentially novel patterns (low pAkt relating to cetuximab sensitivity). Future studies will involve further preclinical validation with different model systems, specifically HNSCC PDXs. Initially, we will focus on the biomarkers identified in this work, with the potential to expand to additional markers from reports in the literature or as the result of in vitro screening processes. The PDX model will provide an intermediate step to further validate in vitro or cell line identified biomarkers prior to assessment in patient tumors which represent a valuable, and limited resource. The ultimate goal is to use these results to aid in the design of a prospective clinical trial, where patients would be stratified by their baseline biomarker profile and prescribed differential treatment based on that profile. Such a trial could eventually lead to the introduction of individualized treatment for HNSCC in the clinic.

## Supporting Information

**Figure S1.** Specificity of antibody reactions for immunohistochemistry and baseline (control) staining intensity. (A) For each antibody utilized for quantitative IHC (EGFR, pEGFR, pAkt, pERK, and ERCC1), control slides from UM-SCC47 xenografts were stained with and without the primary antibody and were subsequently developed with DAB. For each antibody, there is absent brown color (DAB) in the no primary slide while we see presence of this color in the slides with the primary, indicating the specificity of the reaction. (B) Baseline (i.e., control) biomarker staining for each cell line as demonstrated in representative images. Inset numbers represent DAB-MOD values for each stain/slide.

**Figure S2.** Representative ERCC1 IHC images for pre- and posttreatment xenografts in relation to cisplatin response. Images from pre- and posttreatment (4, 24 h) tumors from both a sensitive and resistant xenograft with respect to cisplatin treatment. The row of images obtained from the sensitive xenograft demonstrates no observable changes in nuclear ERCC1 expression between the pre- and posttreatment samples. This same relationship is revealed for the resistant tumor.

**Figure S3.** Representative IHC images for pre- and posttreatment biomarkers related to cetuximab response. (A–D) Images depicting the pre- and posttreatment expression of EGFR, pEGFR, pAkt, and pERK from xenografts that were sensitive or resistant to cetuximab. Examining the rows of the EGFR and pAkt images demonstrates no obvious changes in biomarker expression for either the sensitive or resistant groups. For pEGFR, it appears that at 24 h the expression is decreased for the sensitive group and relatively increased for the resistant cohort. For pERK, there is a decrease in relative expression for both the sensitive and resistant groups at 24 h.

**Figure S4.** Representative IHC images for pre- and posttreatment biomarkers related to radiation treatment. (A–E) Images demonstrating the pre- and posttreatment expression of EGFR, pEGFR, pAkt, pERK, and ERCC1 from representative xenografts that were either sensitive or resistant to radiation. Examining the rows of the EGFR, pEGFR, and ERCC1 images demonstrates no evident changes in biomarker expression post treatment in either the sensitive or resistant groups. For pAkt, it appears that expression is decreased in the sensitive group but increased in the resistant xenograft at 24 h. With respect to pERK expression, there is decreased expression at 4 and 24 h in the sensitive xenograft, while the resistant expression appears unchanged from pretreatment.

**Table S1.** List of cell lines used, sources, and culture conditions. **Table S2.** Primary antibodies used for immunohistochemistry.
