# Supplementary figures and images for: Xenograft assessment of predictive biomarkers for standard head and neck cancer therapies
Source: Cancer Med. 2015 Jan 26;4(5):699–712. doi: 10.1002/cam4.387 (PMC4430263; doi:10.1002/cam4.387)

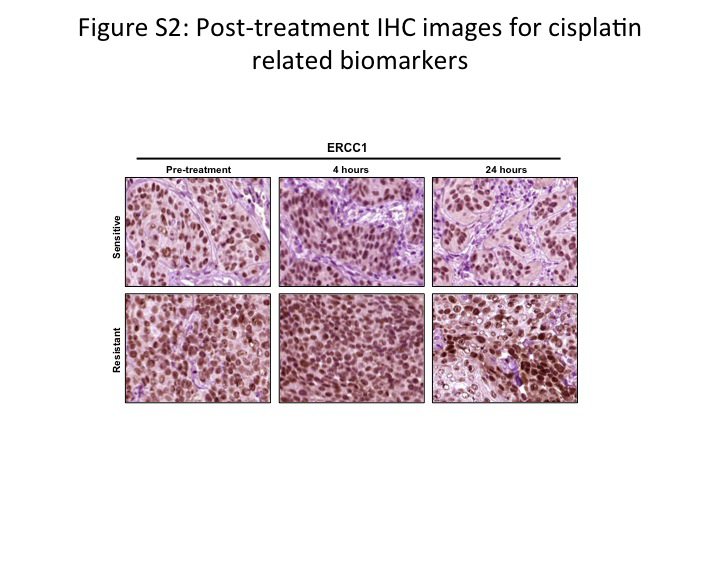

Supplement: Supplementary file 2 — Figure S2. Representative ERCC1 IHC images for pre- and posttreatment xenografts in relation to cisplatin response. Images from pre- and posttreatment (4, 24 h) tumors from both a sensitive and resistant xenograft with respect to cisplatin treatment. The row of images obtained from the sensitive xenograft demonstrates no observable changes in nuclear ERCC1 expression between the pre- and posttreatment samples. This same relationship is revealed for the resistant tumor. [file cam40004-0699-sd2.tif]

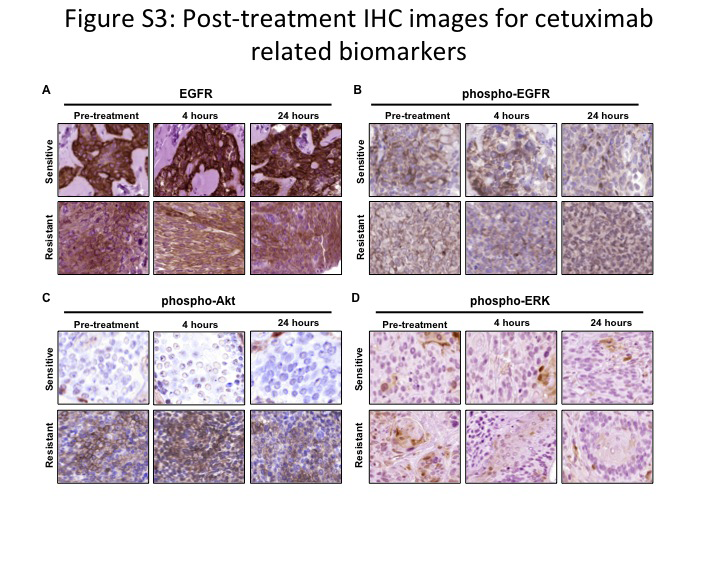

Supplement: Supplementary file 3 — Figure S3. Representative IHC images for pre- and posttreatment biomarkers related to cetuximab response. (A–D) Images depicting the pre- and posttreatment expression of EGFR, pEGFR, pAkt, and pERK from xenografts that were sensitive or resistant to cetuximab. Examining the rows of the EGFR and pAkt images demonstrates no obvious changes in biomarker expression for either the sensitive or resistant groups. For pEGFR, it appears that at 24 h the expression is decreased for the sensitive group and relatively increased for the resistant cohort. For pERK, there is a decrease in relative expression for both the sensitive and resistant groups at 24 h. [file cam40004-0699-sd3.tif]

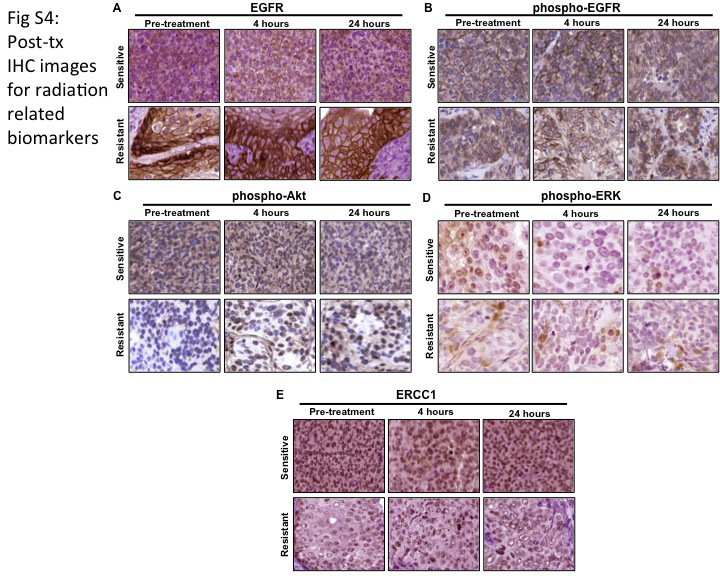

Supplement: Supplementary file 4 — Figure S4. Representative IHC images for pre- and posttreatment biomarkers related to radiation treatment. (A–E) Images demonstrating the pre- and posttreatment expression of EGFR, pEGFR, pAkt, pERK, and ERCC1 from representative xenografts that were either sensitive or resistant to radiation. Examining the rows of the EGFR, pEGFR, and ERCC1 images demonstrates no evident changes in biomarker expression post treatment in either the sensitive or resistant groups. For pAkt, it appears that expression is decreased in the sensitive group but increased in the resistant xenograft at 24 h. With respect to pERK expression, there is decreased expression at 4 and 24 h in the sensitive xenograft, while the resistant expression appears unchanged from pretreatment. [file cam40004-0699-sd4.tif]
