# Supplementary material for: Xenograft assessment of predictive biomarkers for standard head and neck cancer therapies
Source: Cancer Med. 2015 Jan 26;4(5):699–712. doi: 10.1002/cam4.387 (PMC4430263; doi:10.1002/cam4.387)
Supplement: Supplementary file 6 [file cam40004-0699-sd6.doc]

**Supplemental Information**

**Xenograft assessment of predictive biomarkers for standard head and neck cancer therapies**

Andrew P. Stein, BA^1^, Adam D. Swick, PhD^1^, Molly A. Smith, BS^1^, Grace C. Blitzer, BS^1^, Robert Z. Yang, MD^1^, Sandeep Saha, MS^3^, Paul M. Harari, MD^1,5^, Paul F. Lambert, PhD^2,5^, Cheng Z. Liu, MD PhD^4,5^, and Randall J. Kimple, MD PhD^*,1,5^

Departments of Human Oncology^1^, Oncology^2^, Biostatistics^3^, Pathology^4^, and Carbone Cancer Center^5^

University of Wisconsin School of Medicine and Public Health, Madison, WI 53706, U.S.A.

**Supplemental Tables**

**Table S1.** List of cell lines used, sources, and culture conditions.

| **HPV Status** | **Cell line** | **Tumor description** | **Sources** | **Culture condition** |
| --- | --- | --- | --- | --- |
| HPV-negative | UM-SCC1 | Brenner JC et al. Genotyping of 73 UM-SCC head and neck squamous cell carcinoma cell lines.  Head Neck. Apr 2010; 32(4): 417–426. | Dr. Thomas E. Carey, University of Michigan | DMEM with 4.5 g/dL glucose, 10% FBS, 1% hydrocortisone, penicillin (100 units/mL), streptomycin (100 mg/mL) |
|  | UM-SCC22B |  |  |  |
| HPV-positive | UD-SCC2 | Olfhof et al. Load, gene expression and mapping of viral integration sites in HPV16-associated HNSCC cell lines. IJC 2014  doi: 10.1002/ijc.29112 | Dr. Thomas Carey, with permission of Dr. Henning Bier, Technical University Munich, Munich, Germany | DMEM with 4.5 g/dL glucose, 10% FBS, penicillin (100 units/mL), streptomycin (100 mg/mL), |
|  | UM-SCC47 |  | Dr. Thomas E. Carey, University of Michigan |  |
|  | UPCI-SCC90 |  | Dr. Robert Ferris, University of Pittsburgh |  |
|  | 93-VU-147T |  | Dr. Robert Ferris, with permission of Dr. Hans Joenje, VU Medical Center, Amsterdam, Netherlands |  |

**Table S2.** Primary antibodies used for immunohistochemistry.

| **Antibody** | **Abbreviation used in Text** | **Dilution** | **Source** | **Company** | **Catalog #** |
| --- | --- | --- | --- | --- | --- |
| EGFR | EGFR | 1:200 | Rabbit | Cell Signaling Technology, Danvers, MA | 4267 |
| phospho-EGFR (Tyr1173) | pEGFR | 1:500 | Rabbit | Cell Signaling | 4407 |
| phospho-Akt (Ser473) | pAkt | 1:100 | Rabbit | Cell Signaling | 3787 |
| phospho-p44/42 MAPK (Erk1/2) (Thr202/Tyr204) | pERK | 1:400 | Rabbit | Cell Signaling | 4370 |
| ERCC1 | ERCC1 | 1:400 | Mouse | Origene, Rockville, MD | UM570008  (Clone 4F9) |
| Cytokeratin | pan-cytokeratin | 1:1600 | Mouse | DAKO, Carpintiera, CA | M3515 (Clone AE1/AE3) |

**Supplemental Figure Legends**

**Figure S1. Specificity of antibody reactions for immunohistochemistry and baseline (control) staining intensity.** **A)** For each antibody utilized for quantitative IHC (EGFR, pEGFR, pAkt, pERK, and ERCC1), control slides from UM-SCC47 xenografts were stained with and without the primary antibody and were subsequently developed with DAB. For each antibody, there is absent brown color (DAB) in the no primary slide while we see presence of this color in the slides with the primary, indicating the specificity of the reaction. **B)** Baseline (i.e. control) biomarker staining for each cell line as demonstrated in representative images. Inset numbers represent DAB-MOD values for each stain/slide.

**Figure S2. Representative ERCC1 IHC images for pre- and post-treatment xenografts in relation to cisplatin response.** Images from pre- and post-treatment (4 hours, 24 hours) tumors from both a sensitive and resistant xenograft with respect to cisplatin treatment. The row of images obtained from the sensitive xenograft demonstrates no observable changes in nuclear ERCC1 expression between the pre- and post-treatment samples. This same relationship is revealed for the resistant tumor.

**Figure S3. Representative IHC images for pre- and post-treatment biomarkers related to cetuximab response.** (A—D) Images depicting the pre- and post-treatment expression of EGFR, pEGFR, pAkt, and pERK from xenografts that were sensitive or resistant to cetuximab. Examining the rows of the EGFR and pAkt images demonstrates no obvious changes in biomarker expression for either the sensitive or resistant groups. For pEGFR, it appears that at 24 hours the expression is decreased for the sensitive group and relatively increased for the resistant cohort. For pERK, there is a decrease in relative expression for both the sensitive and resistant groups at 24 hours.

**Figure S4. Representative IHC images for pre- and post-treatment biomarkers related to radiation treatment.** (A—E) Images demonstrating the pre- and post-treatment expression of EGFR, pEGFR, pAkt, pERK and ERCC1 from representative xenografts that were either sensitive or resistant to radiation. Examining the rows of the EGFR, pEGFR and ERCC1 images demonstrates no evident changes in biomarker expression post-treatment in either the sensitive or resistant groups. For pAkt, it appears expression is decreased in the sensitive group but increased in the resistant xenograft at 24 hours. With respect to pERK expression, there is decreased expression at 4 and 24 hours in the sensitive xenograft while the resistant expression appears unchanged from pre-treatment.
